# “How can we talk about our struggles?” reflections on researcher wellbeing and recommendations for psychosocial support for researchers in conflict-affected areas

**DOI:** 10.1186/s13031-025-00725-6

**Published:** 2025-11-28

**Authors:** Laura Jeffery, Lucy Lowe, Jean-Benoît Falisse, Rina Ghafoerkhan

**Affiliations:** 1 University of Edinburgh, 15a George Square, Edinburgh, EH8 9LD UK; 2 ARQ National Psychotrauma Centre, Diemen, Netherlands

**Keywords:** Emotionally demanding research, Trauma-informed research, Researcher wellbeing, Psychosocial support, Displacement, Conflict, International collaboration

## Abstract

**Background:**

In recent years, research has revealed the emotionally demanding side of research for researchers (in addition to research participants). This research provides useful guidance, especially for researchers who work with research participants facing difficult situations such as illness, forced displacement, and conflict. Yet, they tend to focus on research projects in the Global North. We explore emotionally demanding research and its impact on researcher wellbeing, and formal support systems and informal mitigation measures in a Global South context marked by conflict and violence and a complex international and interdisciplinary collaboration.

**Methods:**

We interviewed thirteen researchers and one psychosocial staff support specialist involved in a UK Global Challenges Research Fund project on improving healthcare at the intersection of gender and protracted displacement amongst Somali and Congolese refugees and internally displaced people in Somalia, DR Congo, Kenya, and South Africa (2020–2024). The analysis explores the intersections of four recurrent themes: fieldwork conditions, researcher symptoms, support systems, and mitigation measures.

**Results:**

Researching protracted displacement exposed the teams to multiple forms of emotionally demanding work, leading to distress, exhaustion, sleep disruption, guilt, and features of vicarious trauma and moral injury. These impacts were heightened by researchers’ own histories, socio-economic inequalities, COVID-19, and insecurity. Formal psychosocial support was provided, but uptake was low due to timing, online delivery, trust, and unfamiliarity; participants recommended compulsory, continuous, locally grounded, and one‑to‑one options. Informal measures – team debriefings, buddying, adaptive safety practices, and peer support – were widely used and valued. Ethical tensions around aid expectations coming from the research participants persisted.

**Conclusions:**

First, echoing calls for a feminist ethics of care, the article recognises emotional responses as an inherent part of ethical research practice. Second, it articulates the benefits of local wellbeing champions. Third, it highlights that researcher wellbeing support should be a multifaceted and ongoing process rather than a one size fits all once-off event. Fourth, it calls for structural change to recognise emotional challenges as part of research ethics and integrity processes (rather than solely the responsibility of individual researchers) and to budget for devolved and locally appropriate psychosocial support service provision for researchers.

## Introduction and background

Field research is often emotionally demanding, particularly when it focuses on challenges faced by research participants, such as ill-health, forced displacement, and violence. The challenges can be compounded by conducting research in volatile settings, with limited resources, under significant pressure to complete data collection. Such emotionally demanding research requires “mental, emotional, or physical energy” [1] and “has the potential to impact upon the wellbeing of the researcher” [2]. This may, in turn, contribute to high staff turnover and affect the quality and continuity of research in these settings [3]. Afraid of being stigmatised as unsuited to conduct research, researchers may mask their emotions and their experiences of trauma; feminist geographers have criticised the “dominant masculinist rationality” that renders embodied knowledge unreasonable under the framing of professionalism [4–6]). Zwi et al. [7] note that because conflict settings are less likely to have functioning ethical review processes, more responsibility and risk thus falls on researchers themselves. San Roman Pineda and colleagues [5] argue that emotionally burdened researchers may be more likely to make decisions that cause harm to research participants or to limit the scope of the original research design in ways that can compromise the quality of the research. Thus, they argue for a feminist ethics of care as a research practice that simultaneously supports researcher wellbeing, promotes inclusive research environments, protects research participants, and safeguards research quality [5].

Research highlights the importance of psychosocial support for researchers [8]. Supervision, self-care strategies, and organizational accountability are key to mitigate risks such as vicarious trauma and emotional harm during sensitive, often distressing research (for a review of the literature, see [9]). The World Health Organization’s *Ethical and safety recommendations for researching*,* documenting and monitoring sexual violence in emergencies* [10] insists on the importance of strategies for self-care, recommending that: “given the potential for emotional or social harm to those collecting the information, as part of the training programme, team members should engage in candid and honest discussions about this and develop strategies to minimize such effects.” How to do this in practice warrants further attention. Three recent contributions are the Emotionally Demanding Research Network [2], the Researcher Wellbeing Project [11] and the RES-WELL toolkit to support researcher wellbeing [12], which seek to equip researchers to recognise signs in themselves and others with a view to managing and reducing the risks of secondary trauma, deploying coping strategies and grounding techniques, and developing a more reflexive trauma-informed research practice. These initiatives do not, however, engage explicitly with the additional difficulties that emerge in large international collaborations, in particular power and resource asymmetries between partners based in very different contexts. Nor do they directly engage with the additional ethical and wellbeing difficulties posed by research in the context of (post-)conflict and war.

This article examines the experiences of an international and interdisciplinary research team and the extent to which formal psychosocial support and informal support measures met their psychosocial needs in practice. From 2020 to 2024, we ran a UK Global Challenges Research Fund (GCRF) project on improving healthcare at the intersection of gender and protracted displacement amongst Somali and Congolese refugees and internally displaced people (IDPs) in Somalia, DR Congo, Kenya, and South Africa (DiSoCo).^1^ The context in DR Congo (South Kivu) and Somalia (Garowe and Kisamyo) was one of violence and “low-intensity” conflict compounded by extreme poverty and natural disasters [13–15], whilst the research in urban areas of Kenya (Nairobi) and South Africa (Johannesburg) was in deprived and occasionally violent areas [16–18]. The project’s research methods included social connections workshops and household surveys, life history interviews with displaced people, and semi-structured interviews with healthcare providers and other professionals. We anticipated that this would be emotionally demanding research and offered a psychosocial training and support package including an initial 3-hour training workshop and follow-up support from a mental health professional affiliated with ARQ National Psychotrauma Centre (based in the Netherlands). In the event, the project took place during the COVID-19 pandemic, so the training and follow-up support had to be delivered online. During the project, however, we found that this formal psychosocial support system had limitations, and that teams were supplementing this formal option with collective and individual coping strategies. Towards the end of the project, we wanted to capture the valuable suggestions of team members for psychosocial support and training for field researchers. This article starts with methodological and ethical reflections before interweaving our results and discussion in two subsections.

## Methods and ethical reflections

This article is based on interviews with thirteen researchers and one psychosocial staff support specialist involved in our project. The sample was gender balanced (seven men and seven women) with three people in their 20s, four in their 30s, four in their 40s, and three in their 50s. The four co-authors of this article were, respectively, the Principal Investigator, two Co-Investigators, and a mental health researcher. For this article, each of the four co-authors conducted three or four interviews with a mixture of senior Co-Investigators, postdoctoral researchers, and research assistants from the project. One co-author additionally interviewed the mental health professional who provided the psychosocial support training and follow-up support (based at ARQ). Conscious that the power dynamic could compel junior colleagues to participate, we approached our colleagues for these interviews only after the formal end of the funded period in 2024. We conducted all interviews remotely, recorded interviews via MS Teams or Zoom, and corrected the automatically generated verbatim transcripts.

The preamble to our interview guide highlighted that our purpose was to “improve support of researchers’ wellbeing in future projects”. We opened interviews by requesting demographic information about age, gender, and region of work. Thereafter, our semi-structured interview schedule comprised a series of open-ended questions – including some prompts and follow-up questions – about their personal experiences of emotionally-challenging research and their reflections about how best to support researcher wellbeing. The experience of collaboratively designing, conducting, and analysing research on health for DiSoCo meant that interviewers and interviewees for this paper had shared understandings of mental health, idioms of distress, and psychosocial support in the different fieldwork contexts [19]. Our interview structure echoes Smillie and Riddell’s [2] researcher consultation process which asked researchers what experiences had been emotionally demanding, what impacts it had on them, what safeguarding measures and coping strategies had helped, and what else would be useful. Following the interviews, the four co-authors together developed a codebook based on four recurrent themes: fieldwork conditions, researcher symptoms, support systems, and mitigation measures. We then divided each theme into three subthemes – agreeing on an indicative list of examples for each subtheme – and used the open-source online qualitative data analysis tool Taguette [20] to code our interviews according to these twelve subthemes. After coding, we paired themes to structure this article into its two main sections (i.e. on emotionally demanding research and its impacts on researcher wellbeing, and on formal support systems and informal mitigation measures).

We interviewed a minimum of three and a maximum of four people from each of our four study countries (Somalia, DR Congo, Kenya, and South Africa); to safeguard their anonymity, in this article we do not identify them either by institutional affiliation, or by country, or by language (instead translating any quotes from French directly into English), or by gender (instead using “they” as a gender-neutral third-person singular pronoun throughout). Furthermore, our interviewees for this article were all members of research project teams, and most of them had interviewed research participants during their fieldwork; thus, to distinguish *our* interviewees (i.e. those we interviewed for this article) from *their* interviewees (i.e. research participants in the wider project), we refer to *our* interviewees as “researchers” and to *their* interviewees as “research participants”. Limitations of this article include the relatively small number of interviewees, which was determined by the number of researchers involved in DiSoCo. Rich and detailed interviews grounded in the experience of working on this shared project generated reflections and recommendations that we believe are applicable more broadly to others similarly conducting research in challenging settings.

The Researcher Wellbeing Project [12] noted that negative consequences are often compounded for marginalised, precarious, or early career researchers. They interviewed researchers working in the UK, where research culture is a current priority [12]. The question of differentiated consequences does, however, also apply to international collaborations where an additional dimension needs to be accounted for: researchers based in the Global South typically have fewer professional opportunities and financial resources at their disposal than researchers based in Global North institutions. Our interviews with colleagues based in four countries in Africa did not generate direct evidence about precarity, but some of the earlier career researchers we interviewed did mention hierarchies within their research team and power dynamics between teams based in Africa and teams based in Europe. DiSoCo was impacted by the COVID-19 pandemic and the resultant changes to funding, alongside delays in the collaboration agreement and the challenges of implementing administrative changes at the University of Edinburgh. Difficulties including delayed payments and unexpected budget changes exacerbated stress levels and featured in some of the interviews; this article focuses on emotionally challenging research, rather than the administrative challenges of international collaborative research projects (see e.g [21]).

## Results and discussion

### Emotionally demanding research and its impacts on researcher wellbeing

In this section we explore the characteristics of emotionally demanding research and its impacts on researcher wellbeing. Kumar and Cavallaro [1] propose four types of emotionally demanding research experiences: “(a) research on sensitive issues (e.g., violence, abuse, mental health, chronic or terminal illness, death), (b) research similar to personal trauma previously experienced by the researcher, (c) the researcher’s experience of traumatic life events while conducting a study, and (d) unexpected events that arise during research in what was previously *not* identified as a sensitive issue” [1]. All four types resonate very well with our own work on protracted displacement. Moreover, in the project under consideration in this article, researchers might experience multiple types or even all four of these types of emotionally demanding research at once. For instance: researchers were researching sensitive issues relating to protracted displacement (the above-mentioned category a); they often had personal experience of forced displacement (b); they worked in challenging field sites where they were repeatedly exposed to crime and violence (c); and their fieldwork took place throughout the COVID-19 pandemic (d). Smillie and Riddell note that “Exposure to emotionally demanding experiences, material, or processes can impact upon physical and mental health” in myriad ways including secondary and vicarious trauma, compassion fatigue, and burnout [2]. Dickson-Swift et al. [22] note that emotionally demanding research can have negative emotional and physical outcomes for researchers including gastrointestinal problems, insomnia and nightmares, headaches, exhaustion and depression, and threats to physical safety.

#### Emotionally demanding research

Researchers in our project were exposed to narratives of war, abuse, sexual violence, displacement, extreme poverty, and traumatic experiences in the field, and our interview transcripts reveal the significant emotional and psychological toll these had on researchers. Researchers reported distress, emotional exhaustion, physical manifestations of anxiety, feelings of helplessness, and sleep disturbances. Across all interviews, the researchers indicated that listening to difficult stories generated an emotional burden and vicarious trauma (although not necessarily using these terms). These stories often entailed intersecting layers of trauma arising from violence, poverty, and discrimination, to name but a few. One researcher recalled a particularly traumatic event:

“actually one of the participants that my colleague had interviewed died … we think, from the stress of basically trying to survive in the context in which she was in, and I think that really … shows you … how … on the edge people are living in this context. And so you know, having that in your mind and reading people’s stories about that.”

The researchers explained that they expected to hear difficult stories, but also that “it’s very difficult to know how it’s going to be” in practice. Even for seasoned researchers, field encounters often revealed a level of vulnerability or trust from participants that was unforeseen, leading to emotional responses that could not be anticipated. One researcher reflected on this dynamic: “Even though from an academic perspective, you’re seeing in the field the things that aren’t surprising … they’re surprising in the way they manifest or the way that people open up in ways you didn’t think they would”.

#### Impacts of emotionally demanding research on researcher wellbeing

Researchers expressed that they were inadequately prepared to manage the emotional burden when participants shared deeply personal or traumatic stories, leaving researchers grappling with ethical ambiguity and emotional uncertainty. As one researcher put it, “you’ve kind of provoked those kinds of stories or memories. I’m not sure if any training can really teach you to. respond”. Researchers struggled with the emotional toll of conducting and processing interviews while maintaining professional boundaries, which caused researchers to feel helpless, guilty, and emotionally exhausted. One experienced researcher reflected on the emotional toll on researchers of having created spaces where people share “harrowing” stories of having been through “horrific” experiences with researchers who are not trained as therapists. Several researchers reflected on their sense of “helplessness”: that in undertaking research with displaced people they had uncovered trauma by raising difficult – and in some cases re-traumatising – issues, without then being able to help their displaced research participants either psychologically or practically. To be clear, a support and referral protocol was in place for research participants, and here the researchers are referring to their desire to do something by themselves about the general situation – conflict, violence, displacement, and poverty – but not being able to do so. This resonates with the concept of “moral injury”, where field researchers witness severe psychological suffering in participants, whether due to trauma, crises, or injustice, but are constrained by limited resources and research protocol from intervening directly or offering aid [23]. As one researcher put it:

“And this had a detrimental impact on yeah, mental wellbeing throughout our project and our interactions with asylum seekers and refugees it became evident that our role often inadvertently reopened wounds rather than providing healing. Yeah. So these individuals in a state of desperation, often viewed research as approaching them as potential problem solvers. Regrettably our involvement was limited to just listen to the purposes of the research, leaving them with no tangible solution to their ongoing struggles. Yeah, so that’s it. So it’s a pity that we aren’t able to provide any solutions to the problem that these people brought to us, we ended up also being affected by the same problem.”

For many of the researchers, the emotional demands of fieldwork were intensified by their own histories of personal trauma that mirrored the experiences of their participants. The boundary between their perceived need for professional detachment and personal memory blurred [4]. For a researcher who had lost a family member to armed violence, doing the research in potentially dangerous neighbourhoods was often a confrontation with their own history.

Researchers working in their own home or long-term host country had vital contextual knowledge and experience, as well as the resources and relationships necessary to conduct the research. They also described having adapted to the challenges of living and working in conflict areas. When discussing an explosion that occurred during data collection, for instance, one researcher told us:

“To be honest for us like when you grow up and work in [a country affected by protracted conflict and displacement], … you kind of become bit adaptive to that kind of issue. You don’t see it’s much, much big issue. Yeah, it happens. But like you, you don’t stress out when it happens, because it’s usually every day you see the news, you, even if it’s not happening in your area. You will see the news. You will have someone who, you know, has passed away because of the incident. We get bad news every day. So it’s become common.”

This familiarity and adaptation did not eliminate the emotional toll; rather, it added a layer of silent endurance. Many researchers emphasised that, although they were from or lived in the same country as their participants, there were significant socio-economic disparities between them that provoked feelings of guilt, injustice, and moral responsibility. Structural inequalities, the lack of policy implementation, policy failings, and the slow pace of fieldwork compounded researchers’ despondency. One researcher said that their team had been surprised by the extremely poor living conditions of participants when visiting a site for the first time, despite living in geographic proximity. A researcher in a different team poignantly asked:

“Why this injustice? Why this inequality? What have I done more to deserve my little life? When we returned from the surveys, we came back with several questions and hypotheses, without knowing where to find answers.”

Several researchers reported a sense of “guilt” that they and their families were safe and had access to healthcare. Repeated exposure to suffering, without the capacity to meaningfully change participants’ circumstances, created a sense of emotional fatigue and moral injury. In this context, it’s unsurprising that the researchers often reported a sense of helplessness:

“the desperation … we know that the health system does not work, but when you have children affected … this kid … who was in … constant pain. And there’s nothing we could do, I mean I tried.”

Despite working for an NGO that supports children and having connections to a local hospital, their efforts to get medical care for the child were unsuccessful. They concluded that their sense of helplessness and guilt was compounded by the “double burden” of being a national of the country where they were conducting this challenging research. For several researchers, the stress of research was compounded by unanticipated events that arose during research and by acute personal crises when researchers experienced their own new traumatic events during their fieldwork. In South Africa, and to a lesser extent Kenya, the unpredictability of the COVID-19 pandemic complicated their work and amplified mental strain and personal vulnerability in an already challenging environment. One researcher reported that “the violence, particularly gender-based violence, became rampant within the Congolese community, leading to the disruption of many families”. Researchers in South Africa also described the fear of infection while travelling to their field site, and the emotional weight of contracting COVID-19 themselves. Researchers were ambivalent about switching to online interviews: on the one hand, distance provided protection (and made some of the initial contacts as easy as scheduling a call), but on the other hand, it damaged rapport and compounded their sense of helplessness. These challenges and concerns were not shared by colleagues in Somalia or DRC, where the pandemic did not significantly impact the research. Beyond COVID-19, which mostly featured in our interviews with researchers in South Africa, many researchers reported having had to navigate security threats, including from armed groups such as Al-Shabaab in Somalia. Two reported that researchers were perceived as wealthy outsiders and thus vulnerable to crime: “Sometimes the fact that you are from an institution of higher learning, the idea is that you have money, it also adds to that risky factor”. Additionally, the focus of the research on a specific group of displaced people introduced an added layer of unsafety, vulnerability, and ethical dilemma for the researcher, as illustrated in the following excerpt:

“We were more focused on Congolese refugees, but you see there were some other nationalities who wanted to be interviewed, because they knew there was some money and foods that was given. So at some point you are being harassed, and they are, like, you guys are not good. Why are you guys … discriminating us, as where you only focusing yourself on Congolese? As you know we are all suffering. We are just like them, we are also suffering.”

The researchers’ struggles with emotional burdens and ethical dilemmas underscore the need for better mental health support for researchers, safety measures, and ethical considerations of conducting research in similar settings.

### Formal support systems and informal mitigation measures

The project team members were already experienced displacement and health researchers and therefore anticipated the potential impact of data collection on researcher wellbeing. As such, psychosocial training and support were included in the project design from the outset. This aligns with concurrent multidisciplinary scholarship that calls for further attention to the emotional wellbeing and safety of researchers. Silverio et al. [24] suggest a series of practical principles for supporting qualitative researchers: training and induction; appropriate scheduling of data collection; the “buddy system”; effective debriefing; reflective diaries or journals; individual and team supervision; charitable input and support; and formal psychological support. Williamson et al. [25] submitted to their Research Ethics Committee a researcher safety protocol that specifically mentioned emotional safety and provided for debriefing, emotional aspects of the work as a standing item on team meeting agendas, and counselling services. Skinner et al. [11] suggest each researcher should develop their own personal wellbeing plan (which we did not consider prior to our own GCRF project). Their Researcher Wellbeing Project distinguished between formal coping mechanisms (such as wellbeing plans, teamwork, clinical supervision, counselling) and informal self-care (whether individually or with colleagues or friends). We similarly distinguished between formal and informal coping mechanisms.

#### Formal support systems

In terms of formal training and support, the Researcher Wellbeing Project concluded that “Ideally, institutions that undertake and fund research on emotionally challenging topics need to put plans in place to develop a well-funded strategy focused on *prevention* of and *provision* for distress and secondary trauma linked to such research” [11]. The RES-WELL toolkit articulates that “Supporting researcher mental health and wellbeing requires a multipronged approach from funders, institutions, senior staff managers, supervisors, and researchers themselves” [12]. These recommendations resonate with the design of our project’s package of training and support. Our project’s formal psychosocial support (PSS) training consisted of a 3-hour workshop delivered by an ARQ mental health professional. All project partners were offered PSS training, and all but one team took up the offer. We originally intended in-person training with all teams together, but in practice this was impossible due to the international COVID-19 travel restrictions that coincided with the launch of the project. ARQ adapted the training and the ARQ facilitator delivered the workshops online to each team separately, in English (with a member of the Edinburgh team providing French interpretation for those based in DR Congo). The ARQ facilitator provided basic staff care training, which included discussions on signs and symptoms of distress, how to recognise stress in oneself and others, and possible coping mechanisms. The facilitator asked the groups to identify key stressors in their field sites and reflect on how they have managed them in the past, before asking how they might consider addressing them in future.

The PSS training was intended to generate an open space for the research teams to discuss the likely possibility of stress during the research and to collectively consider coping strategies that would allow them to care for themselves and other members of their team, as well as making them alert to the potential of causing distress to participants. As the ARQ facilitator explained,

“We didn’t frame it as we’re going to talk about the troubles you are having, or any mental health difficulties you’re experiencing. But it was really like, ‘this is a new experience for many of you working in this kind of research setting with these beneficiaries… Let’s see what kind of things might come up with this work.”

They discussed cultural idioms and signs of extreme stress in their communities to tailor the discussion to the local context and build on existing knowledge in the teams. Online facilitation overcame geographic distances and travel restrictions but, during interviews, the ARQ facilitator and the researchers emphasised the challenges of online workshops. Variable internet connections frequently caused problems, and in some cases it was only possible to participate with cameras switched off. This made it difficult to build rapport within the group and to discuss sensitive topics. The ARQ facilitator, based in the Netherlands, reflected,

“Was it appropriate in the sense that could people relate to me enough, or did it feel like I’m talking from here, and their reality is here? […] I think sometimes I felt like a fish out of water. I was wondering like, ‘I hope this is landing well’, you know, like I’ve done my research, I’m trying to look at what is specific to this area and talk to people. But it’s sometimes hard to know still, afterwards… Are there certain things that I totally missed because I’m just not aware of it?”

In a similar vein, the facilitator also raised the benefits of local, or at least regional expertise; for example, when one team asked for advice on how to respond to participants who express suicidal ideation, the facilitator sought advice from a colleague with expertise in the topic, who was also from the region of our research and had experience working in refugee contexts. The ARQ facilitator noted the benefits of having an Edinburgh team member interpret during the training with the DR Congo teams with whom he already had an established research relationship.

“He was there with me, and I think that was helpful, because he already had relationships with some of the people from the team, and I think that facilitated it a lot, in fact, I think that created some more trust in me as well, even though it was me doing most of the facilitation, but the fact that he could introduce me to it made a big difference.”

On the other hand, some researchers in another group did not appreciate the presence in their PSS workshop of an Edinburgh team member who they did not yet know well:

“How can we talk about our struggles when the person who is … handling the research is in the call? Even when he was not there, he would be there in the beginning and then log out… Yeah, so it felt a little bit invasive. Like he was invading our like psychosocial support group.”

The researchers had varying levels of previous PSS training. Some had worked for large humanitarian or development organisations, where they had received staff training, others had received basic stress-management training in school or college, and some had not previously had any at all. One researcher told us,

“It was good because I’ve never had a training like that before to start off. Like mentally preparing someone for what they’re going to see or what they’re going to hear is really good. Especially for people who are doing research with refugees for the first time. Because they’re just a special kind of group that just go through so much.”

Interviewees who undertook the project’s PSS training reported that they had found it very helpful – two researchers spontaneously mentioned appreciating the breathing exercises – but admitted that they had not actually managed to deploy these skills during stressful episodes in the field, when they found it hard to recall what they had learned. A researcher whose team had attended follow-up group sessions with the project’s remote PSS support service reported that they did not feel free to truly open up about their experiences in the field, and recommended one-to-one check-ins after data collection “because sometimes people don’t reach out unless someone else reaches out”. One project team reported that they didn’t do the project’s PSS training not because they had actively opted out, but because they lacked capacity at the time it was offered.

Despite the provision of PSS across the teams and throughout the project, there was very little uptake beyond the initial basic training. The ARQ support package included an offer of follow-up consultation after the PSS workshop, but only one team made use of this offer. The ARQ facilitator said,

“That makes me wonder if there were more things going on. But either (a) they were just really doing great. They were taking great care of each other already. It wasn’t a big deal, or (b) if they forgot about me, or I wasn’t on their radar, or (c) they didn’t think that it was the appropriate form of support.”

One researcher explained that junior researchers “wouldn’t reach out to [the facilitator] and be like, you know what, I need some kind of support”. They explained that this was firstly because they had never previously sought support for workplace distress, and secondly because they only knew the facilitator as a remote individual on a group Zoom call, who would not have sufficient understanding of their experiences. Members of several project teams indicated that the fact that the project PSS was based remotely (in the Netherlands) made it difficult for in-country team members to imagine its usefulness. In relation to PSS training and support alike, mitigations spontaneously suggested by two researchers included making PSS activities both compulsory and continuous; several researchers suggested that formalising and normalising PSS training and support for all team members – regardless of experience and seniority – could also have a positive knock-on effect for junior researchers.

The researchers we interviewed were also not aware of anyone seeking out other formal support, such as speaking to mental health professionals. Two partner institutions also offered mental health support for staff, but the researchers we interviewed had not used it to support them during this research, and some staff at those institutions were not aware of those services. One researcher reported having experienced symptoms, but said there was no support available,

“it was so traumatising and difficult to sleep in the night, because to be honest it was coming back to me and giving me a lot of headache and unfortunately there was no particular formal support. I had to deal with this situation in my own way, for these stories that were very, very affecting, I just have to talk to some friends who offered me a bit of support, and some informal counselling service.”

One researcher, echoing the experiences of many DiSoCo research participants, mentioned the lack of mental health services in their country in general, and the reluctance to take up what little provision there is. Although some DiSoCo participants discussed the stigma surrounding mental health in our fieldwork contexts [19], our researchers did not indicate that they felt such stigma and were generally comfortable discussing the need for psychosocial support. Some researcher noted that the COVID-19 restrictions and resulting delays meant that they had started fieldwork before the training was delivered, and that the training therefore came too late to adequately prepare them.

#### Informal mitigation measures

Several researchers reflected on their personal exposure and the exposure of their research participants to COVID-19 and on deliberations within their respective teams about whether, when, and how to move to online interviews and/or resume in-person fieldwork. As noted above, this primarily affected researchers in South Africa. Our project’s fieldwork period also coincided with escalation of violence in some of our potential field sites; one such project team chose relatively “safe areas”, paired male and female researchers, and shared mobile numbers: as one team leader put it, “we chose our safety first”. This team considered hiring a security detail but decided that this would draw attention and elicit suspicion. Nevertheless, this team had to leave a field site suddenly after hearing an explosion nearby; they returned to their field site only after their community gatekeepers had explained that this had been a targeted bomb attack and that the research team was not the target. For members of this team, accustomed as they were to living in a context of volatility, “you kind of become a bit adaptive to that kind of issue”, but after this episode the team did check in on one another and later had a more formal debriefing. The research team debriefed routinely to ascertain whether difficult fieldwork had “taken a toll” on any team members who might therefore need to decompress, for example by being assigned desk-based work instead of more data collection the following day.

In another field site, researchers reported having been advised to gain access via community gatekeepers, avoid isolated areas, and instead conduct research interviews in quiet corners of public spaces. Nonetheless, several researchers were exposed to violence and crime during their fieldwork. Researchers who conducted fieldwork on their own rather than in groups reported having been robbed (resulting in a decision to travel via hire car rather than by public transport), having been verbally abused in a crowd (resulting in a decision always to be accompanied by a community gatekeeper), and having once felt so unsafe during an interview in a public space that they had terminated an interview midway (resulting in a sense of having missed key data).

In less acutely violent contexts, such as parts of Kenya and DR Congo, the emotional toll was somewhat moderated by the ability to return home after fieldwork. “We used to go back home at the end of the day, which was good”. Another team found that working far from home, which required them to stay together in a hotel, created the space for valuable informal nightly debriefing sessions that did not happen when conducting research closer to home. In both contexts researchers remained emotionally engaged, experiencing distress not only from the content of interviews but from the broader recognition of structural inequality and their own limited power to intervene. Several interviewees emphasised the importance of peer debriefings and psychosocial support within their teams, which varied in scale and formality, including informal one-to-one and group conversations and more formal debriefing meetings. Team debriefings were frequently cited as a useful tool that researchers had learnt during this project, with some mentioning that they had incorporated them into subsequent projects. Debriefings had a dual function as a methodological tool to review and if necessary adjust fieldwork plans, and as a psychosocial tool where researchers could process experiences, address emotional or psychological impact, and strategise mitigation of stress and trauma. Debriefings as a multipurpose tool allowed space to acknowledge and address the impact of the research in a manner that seemed more intuitive to the teams as a standard research practice, rather than an additional standalone psychosocial activity imposed by the international team.

Additionally, some researchers coped with distress by making sense of suffering through cultural and religious perspectives or by emotionally distancing themselves. Some found that remaining occupied with work helped mitigate distress, while others developed long-term commitments to improving healthcare and social infrastructure. One researcher reported that their shock at the poor conditions in a rural health centre had first prompted them to initiate community and diaspora fundraising initiatives to improve healthcare facilities in their home country. They noted, however, that:

“you have to be careful because … [if the research participants] know that you are providing a support, then it may deviate your goals for collecting that kind of information and you may attract a lot of people and that will be another challenge.”

Researchers went into fieldwork equipped with lists of support services available to displaced people in that area and with money to compensate research participants for their time, but they reported sometimes feeling compelled to top up the compensation: “It really affected me to the point I had to give her extra money”. Researchers reported mixed feelings about the stock response to their research participants that, as one researcher put it, “this data is important to us because it can help us like inform policy makers of what is happening on the ground”. One researcher reported having been invited to give a talk about research findings to a diaspora organisation, noting that:

“We could do a little more to ensure that those with decision-making power are informed of the results of our research. But the problem is whether it will be a priority for them.”

## Conclusions and recommendations

During the interviews, several researchers gave detailed responses to questions on what they thought would be useful to be provided for researchers working in difficult fieldwork conditions and/or on difficult topics. They emphasised the need for preparatory training (on cultural sensitivity, trauma-informed research practices, safety protocols, and ethical considerations) and for information about local support resources for researchers and participants to be available during this initial stage (including mental health support services, safety equipment, transportation, and accommodation if necessary).

Research teams navigating sensitive and traumatic topics face not only the ethical complexities of safeguarding participants, but also significant and under-acknowledged risks to their own wellbeing. Scholarship has criticised dominant research cultures that downplay or even stigmatise researchers’ emotional distress and thus occlude the embodied, affective labour inherent in such research [4, 5]. As argued by Smillie and Riddell [2] and Zschomler et al. [12], this poses a dual threat, which is compounded in contexts marked by violence, displacement, and precarity: on the one hand it puts the personal welfare of researchers at risk and on the other hand it challenges the rigour and ethical integrity of social science research. Psychosocial support is, therefore, key.

The limited uptake of remote, externally facilitated support, which was developed for our international project, highlights a recurrent theme in the literature; top-down approaches often fail to resonate, particularly in cross-cultural settings [9, 12]. The support mechanism, however, had the key advantage of initiating discussions and reflections. In practice, research teams developed their own support mechanisms. Our research suggests four avenues for future research and action, which should be taken not as firm prescriptions but rather as invitations to develop bespoke co-learning practices necessitated by the contexts in which projects like DiSoCo operate, which almost unavoidably entail substantial power asymmetries and different cultural understandings. Co-learning in and of itself constitutes a useful step towards shifting power dynamics and increase the wellbeing of national researchers.

First, we echo calls for a feminist ethics of care [5] that recognises and legitimises emotional responses as central to ethical research. Any project should begin by acknowledging the emotional burden of research; this is best achieved by taking concrete measures and establishing procedures. These may not be perfect or substantially improve wellbeing in and of themselves, as in our case study, but they help with “normalising” and “depathologising” emotional distress; emotional difficulties are to be expected, and they can be talked about. In international projects, this feminist ethics of care should include efforts to address equitable international research partnerships head-on; supporting wellbeing requires not only offering psychosocial resources, but also ensuring material support, fair compensation, and meaningful inclusion in project governance and decision-making.

Second, in collaborative projects, we recommend identifying a wellbeing champion for each fieldwork team who can co-facilitate PSS training and allow for culturally grounded forms of support, thus potentially enhancing both the uptake and effectiveness of psychosocial support. The local wellbeing champion could then act as an initial point of contact should any issues arise, with a PSS professional acting as “back up” supervisor. Our evidence points to the importance of local contextualisation, trust-building, and the proactive integration of wellbeing strategies into the fabric of research projects.

Third, in line with the literature [11, 24], trauma-oriented psychosocial training and support should not be viewed as a one-off exercise, but rather as integral to all stages of a research project. This article highlights a changing environment that may necessitate adjustments to support. Participants suggested that better use could have been made of local and international teams to provide a peer support network, where researchers could share experiences, seek advice, and provide emotional support. Support should, ultimately, be seen as a combination of different strategies, e.g. training, peer support, structured debriefing, crisis management plans (to handle emergencies, security threats, or situations requiring immediate intervention), and more proactive measures reaching out to researchers such as individualised one-to-one check-ins. Our research showed that some found solace in informal peer support, but others felt isolated and wished for more formal support such as regular debriefing sessions, psychological assistance, and consistent team meetings. This multiplicity of support mechanisms acknowledges different profiles of researchers and addresses different types of trauma and emotional challenges.

Fourth, responsibility for researcher wellbeing does not lie solely with researchers [12]. Journals, funders, and institutions require evidence of ethics protocols that focus on research participants, but should also consider researcher wellbeing, including a budget for devolved and locally appropriate psychosocial support service provision for researchers. In the context of conflict and health research, these recommendations are not options, but essential safeguards for the quality, integrity, ethics, and sustainability of our collective work.

## Data Availability

The datasets generated and analysed during the current study are not publicly available due to the small sample size and because interviewees are members of a named research team. Interview transcripts cannot be shared without risking identification of the interviewees, which would compromise individual privacy.

## Footnotes

1
https://displacement.sps.ed.ac.uk/.
